# Relationships Between Self-Report Hearing Scales, Listening Effort, and Speech Perception in Cocktail Party Noise in Hearing-Aided Patients

**DOI:** 10.3390/audiolres15050113

**Published:** 2025-09-08

**Authors:** Annie Moulin, Pierre-Emmanuel Aguera, Mathieu Ferschneider

**Affiliations:** 1 C. Bernard University, Lyon 1, Inserm U1028, CNRS UMR 5292, Lyon Neuroscience Research Center, Perception, Attention and Memory Team (PAM), F-69 000 Lyon, France; mathieu.ferschneider@inserm.fr; 2 INSERM Rhône-Alpes Directorate, Information Technology Department, F-69 000 Lyon, France; 3 Audition Conseil, F-69 000 Lyon, France

**Keywords:** auditory questionnaire, ageing, hearing loss, rehabilitative audiology, self-report assessment, speech-in-noise perception, closed-set tests, hearing aids, listening effort

## Abstract

Background/Objectives: Potential correlations between the scores of self-report questionnaires and speech perception in noise abilities vary widely among studies and have been little explored in patients with conventional hearing aids (HAs). This study aimed to analyse the interrelations between (1) self-report auditory scales (the 15-item short-form of the Speech Spatial and Qualities of Hearing Scale (15iSSQ) and the Extended Listening Effort Assessment Scale (EEAS); (2) speech perception in cocktail party noise, measured with and without HAs; and (3) a self-assessment of the listening effort perceived during the speech in a noise-perception task (TLE) in hearing-aid wearers. Material and Methods: –Thirty-two patients, aged of 77.5 years (SD = 12) with a mean HA experience of 5.6 years, completed the 15iSSQ and EEAS. Their speech-in-babble-noise perception thresholds (SPIN) were assessed with (HA_SPIN) and without their HAs (UA_SPIN), using a four-alternative forced-choice test in free field, with several fixed Signal to Noise ratios (SNR). They were asked to self-assess their listening effort at each of those SNRs, allowing us to define a task-related listening-effort threshold with (HA_TLE) and without HAs (UA_TLE), i.e., the SNR for which they self-evaluated their listening effort as 5 out of 10. Results: 15iSSQ decreased as both HA_SPIN (r = −0.47, *p* < 0.01) and HA_TLE increased (r = −0.36, *p* < 0.05). The relationship between 15iSSQ*Speech* and UA_SPIN (and UA_TLE) showed a strong moderating influence by HA experience and HA daily wear (HADW), explaining up to 31% of the variance. 15iSSQ*Quality* depended on HA SPIN and HA_TLE (r = −0.50, *p* < 0.01), and the relationship between 15iSSQ*Quality* and UA_TLE was moderated by HADW. EEAS scores depended on both HA experience and UA_SPIN, with a strong moderating influence by HADW. Conclusions: Relationships between auditory questionnaires and SPIN are strongly moderated by both HA experience and HADW, even in experienced HA users, showing the need to account for these variables when analysing relationships between questionnaires and hearing-in-noise tests in experienced HA wearers.

## 1. Introduction

Using data from the 2021 Global Burden of Disease study, Dong et al. (2025) [1] report an increase of more than 108% for the prevalence of age-related hearing loss between 1990 and 2021, and a further increase of 49% between 2022 and 2050, highlighting the importance of this global public health issue. It is worth noting that these numbers are an underestimation of hearing-loss prevalence, as they do not include causes such as meningitis, chronic otitis media, nor congenital factors. Due to the now established links between untreated hearing loss and cognitive decline [2,3,4,5], especially in the elderly population, early intervention by hearing rehabilitation is of major importance [6,7], often contributing to better hearing-aid use [8]. However, even when financial barriers are levied, there is still a relatively low level of hearing-aid (HA) fitting comparatively to the prevalence of hearing loss [9,10,11]. In addition, between 1% and 5% of people (up to 22% in some studies [12]), fitted with hearing aids, do not use them [13]. One way to address the causes of this problem is to assess how people feel about their hearing deficits in everyday life. It has long been established that behavioural audiometric tests (pure-tone audiometry, speech audiometry, aided thresholds) provide only a partial account of the hearing deficits experienced in daily life, or of the benefits of HAs [14,15,16]. This observation, repeatedly confirmed, led to the creation of one of the first auditory questionnaires, the “scale for assessment of hearing” [17], which has been purposely made, according to its creators, to depict everyday hearing experiences “likely to be encountered by most persons living in an urban environment”. Since then, the development of questionnaires, or patient-related outcome measures (PROMs) has been an ongoing process [18,19] and still continues today (e.g., Heinrich et al., 2019 [20], Smith et al., 2024 [21]). These PROMs remain an essential tool to assess hearing disabilities and perceived HA benefits in everyday life. Indeed, one of the most frequent complaints by patients is the challenge represented by communication in various noisy environments. Yet, correlation studies between the various speech perception in noise laboratory tests and hearing scales, remain relatively sparse [22,23], even for one of the most widely used scales in the current international literature: the Speech, Spatial and Qualities of Hearing Scale (SSQ) [24,25], since translated and adapted to multiple languages [26,27,28,29,30,31,32,33]. This scale, composed of 49 items grouped into three different subscales [34], describes everyday communication situations for which the patient is asked to self-assess his or her abilities from 0 (cannot do it) up to 10 (can perfectly do it). The full SSQ form can be quite taxing for some patients [35]; thus, several short forms have been developed: a 5-item SSQ mostly for screening purposes [26], a 12-item SSQ for a global SSQ score [36,37], and a 15-item SSQ (15iSSQ) designed to reflect the specificities of the three initial SSQ subscales [38]. Correlation studies between SSQ and Speech in noise thresholds (SPIN) have been most often focused on normal-hearing or near-normal-hearing populations [39] or individuals with moderate, unaided hearing loss [40,41,42,43], and on individuals with cochlear implants [44,45,46,47,48]. Supplemental Table S1, adapted and updated from Stenbäck et al. (2023) [22], summarises studies reporting some form of correlation between SSQ and SPIN, in various populations, excluding cochlear implant studies. The correlations obtained are all fairly weak to moderate, with correlation coefficients ranging from 0.20 to 0.5 when statistically significant. The strength of such correlations depends on the SPIN tests used [22]. In 44 persons with hearing impairment, aged 50 to 74 years, a significant decrease in SSQ scores as the digit triplet test in the speech-shaped noise threshold increased was observed, but no relationship with an adaptive sentence-based test was obtained in the same population [49]. Similar differences in correlations depending on the SPIN test used were observed in a noise-exposed, near-normal hearing population [40]. Those apparent contradictions in the literature regarding the strength of correlation between hearing questionnaires and SPIN tests can, to a large extend, be attributed to methodological differences in the tests used [49]. Indeed, top-down contextual influences, which are of increasing importance as the complexity of the signal increases (from syllables to full sentences) can increase unwanted variability and decrease reproducibility in tests scores [50,51]. The other major unknown in the relationship between PROMs and laboratory tests is how patients self-assess their hearing abilities, especially when they are HA wearers. The decrease in SSQ scores with increasing hearing loss is steeper for populations with unaided hearing loss than for populations with aided hearing loss [34,35,52], suggesting that hearing-aided patients, as instructed, self-assess their hearing deficits in the situation where they wear their HAs. However, the narrative process around HA fitting can be more important, yielding significant differences in three outcome measures, than acoustical differences between HAs, in experienced HA users [53]. More recently, Wu et al. (2020) [54] showed that several retrospective auditory PROMs were less sensitive in detecting HA benefits difference than ecological momentary assessments, which asks patients to self-assess their experience directly in the actual natural environments they are in, suggesting a recall bias when using PROMs.

Studies reporting correlations between PROMs and SPIN in wearers of conventional HA are extremely scarce [22,55]. A significant increase in SSQ scores, and all three subscale scores, were observed as SPIN improved in a population of patients with unilateral hearing losses [55]; in a large population of HA wearers, however, no significant correlations between SSQ scores and several SPIN tests were obtained [22].

In the present study, we aim to further elucidate the relationships between PROMs and SPIN in experienced HA wearers. To this aim, we chose a closed-set speech-in-babble-noise test, adapted from the British Four Alternative Feature test [56,57] and highly reproducible [58]. This test was presented in HA wearers in 2 conditions: patients wearing their HA (hearing-aided condition) and patients not wearing them during the test (un-aided condition). For both conditions, the patients were asked to self-assess their own listening effort deployed during the speech in the babble-noise test. Lastly, patients were invited to answer two PROMs: they were asked to self-assess the listening effort they deploy in everyday life situations when wearing their HA, in both quiet and noisy environments, using the extended version of the effort assessment scale (EEAS) [59,60]. They were also asked to assess their hearing abilities using the 15iSSQ, which has been shown to be more sensitive to hearing deficits than the full SSQ form or the other short forms [38]. The aim of the study was to analyse the correlations (1) between the PROMs scores and speech-in-babble-noise thresholds, measured with and without HAs, and (2) between PROMs scores, particularly the EEAS scores, and task-related listening effort obtained during the SPIN tests.

## 2. Materials and Methods

### 2.1. Population

Data were collected in a population of patients with symmetric sensorineural hearing loss, equipped with bilateral conventional hearing aids (i.e., air conduction, non-implanted hearing aids (HAs)), and recruited from a hearing-aid clinical practice during their normal follow-up schedule. The hearing aids were of different trademarks and were fitted and tailored to each patient’s needs using an audiologist-driven approach, based on the National Acoustics Laboratories’ non-linear (NAL-NL2) fitting procedure [61,62], the manufacturer’s proprietary software when relevant, and the patient’s feedback. The HA daily wear (HADW), in hours per day, was obtained from the HAs’ data logging facilities.

Patients under guardianship were not included in the study. Patients had to be free of neurological disease, particularly dementia, be French native speakers, be able to read and understand French fluently, and having had HAs for more than 18 months. A total of 32 patients (19 women), with an HA experience ranging from 1.5 to 35 years (median: 6 years, mean: 5.6 years, SD = 7.0) and an average age of 77.5 years (SD = 12.3) were recruited. Pure-tone audiometry was performed in a sound-proof booth, at octave frequencies between 250 Hz and 8 kHz, using an Aurical Astera Audiometer (Otometrics, Natus neuro France). The aided pure-tone thresholds were obtained at the same frequencies using warble tones presented via loudspeakers. Table 1 shows patient characteristics. All patients gave informed consent and the study was approved by an ethical committee (“comité de protection des personnes”, CPP Ile de France II, number ID-RCB 2021-A00076-35, date 21 April 2021).

### 2.2. Speech Perception in Cocktail Party Noise

Speech perception in 16-different-voices (8 female) cocktail party noise was performed using a 4-alternative forced-choice test, initially modelled on the Audimots [63], itself a French language adaptation of the British Four Alternative Feature test [56,57], further adapted for young children [58]. This method was chosen to minimise top-down contextual influences that add variability in open-set tests and to be able to repeat the test without a learning effect [58]. The patients were invited to hear a word in continuous babble noise, and to choose, on a computer tablet, the picture corresponding to the word heard among the 4 different pictures. There was no feedback and the click on the tablet triggered the following screen of 4 pictures. Twelve different screens of 4 pictures were used and presented in a pseudo-randomised manner, so that the same 4 pictures were never presented one after another, and each one of the 48 words was presented only once. To minimise variability due to different types of hearing devices, the patients were tested using one of the most challenging conditions: the signal and noise were collocated and presented through two loudspeakers, positioned in front of the patient at a 60° angle from each other. The signal level was adjusted so that the patient could achieve a score above 95% in silence. At this signal level, the cocktail party noise intensity was then varied to obtain between 4 and 6 different signal-to-noise ratios (SNRs) ranging from −6 to +18 dB, separated by at least 3 dB. For each fixed SNR, the babble noise was continuously played and started at least 20 s before the first word that was voluntarily triggered by the patient. At each of these SNR levels, the patients were asked to rate the listening effort they had to exert, on a visualanalog scale from 0 (no effort) to 10 (maximum effort), according to similar procedures as in Johnson et al. [64] and Holube et al. [65]. The percentage correct responses and the rating of listening effort allowed us to build psychometric functions, to obtain the speech-in-noise threshold (SPIN) in dB SNR, and the corresponding task-related listening effort threshold (TLE). Each patient performed the test under 2 conditions: with their hearing aids and without their hearing aids, the first condition being chosen at random. Thus, for each patient we obtained an unaided SPIN threshold (UA_SPIN), an unaided task-related listening effort (UA_TLE), and the corresponding hearing-aided SPIN threshold (HA_SPIN) and hearing-aided task-related listening effort (HA_TLE). These TLEs represent the thresholds (in dB SNR) at which each patient rated his/her listening effort as 5/10.

### 2.3. Patient-Reported Outcome Measures (PROMs)

The French version of the 15-item Speech Spatial and Qualities of Hearing Scale (SSQ) short form, 15iSSQ [38], and the French version of the Extended Effort Assessment Scale (EEAS) [59] were presented to each patient, in pen and paper format. The order of the two PROMs was randomised and the questionnaires were completed during the same test session as the speech-in-noise tests. The patients were specifically instructed to complete the PROMs as if they were wearing their hearing aids in the everyday situations depicted in each item.

The 15iSSQ short-form was chosen for its brevity, better sensitivity to hearing deficits, and better specificity of each of its 3 subscales, by comparison with the other short-forms and the SSQ full form [38]. For each item, a situation often met in daily life is described (e.g., “*You are in a group of about 5 people in a busy restaurant./…/ Can you follow the conversation?”*) and the patient is invited to rate, on a graded scale from 0 (cannot do the task depicted) to 10 (can perfectly do the task depicted) his or her own hearing abilities. The SSQ is a self-assessment of hearing abilities in the patient‘s daily life, with 3 aspects: SSQ*Speech* relates to speech perception mainly in noise; SSQ*Spatial* explores spatial hearing, sound localisation, and hearing asymmetry; and SSQ*Quality* assesses clarity and discriminability of sounds.

The EEAS was built based on the effort assessment scale [60] and uses 10 items, 3 of which depict situations in hearing in quiet situations and the other 7 items depict situations about hearing in noise. For each situation, the patient was invited to rate the listening effort he/she feels, on a graded scale from 0 (no effort) to 10 (maximum effort). The EEAS score is calculated as the average of the 10 items, with an EEAS*Quiet* subscale averaging the 3 items pertaining to listening in quiet, and an EEAS*Noise* subscale that averages the items pertaining to the noisy situations [59].

### 2.4. Statistical Analysis

Scores of each subscale for 15iSSQ and EEAS were calculated per patient. Missing data on one item were replaced by the average of the other items within the same subscale. A total of 16 EEAS item scores (out of 320) and 12 15iSSQ items (out of 480) were hence replaced. As the 15iSSQ scale has been shown to be a 3-factor scale, with each subscale exploring a different aspect of hearing [38], correlations between the 15iSSQ and SPIN tests were assessed for each subscale. For ease in comparing figures, a colour scale was adapted so that light colours are associated with better hearing abilities and least effort, whereas dark colours are associated with poor hearing abilities and important listening effort.

Statistical analyses were performed using R software version 4.4.2. (31 October 2024) and figures were drawn using ggplot2 version 3.5.1. Differences between correlation coefficients were analysed using Steiger’s test [66].

**Figure 1:**
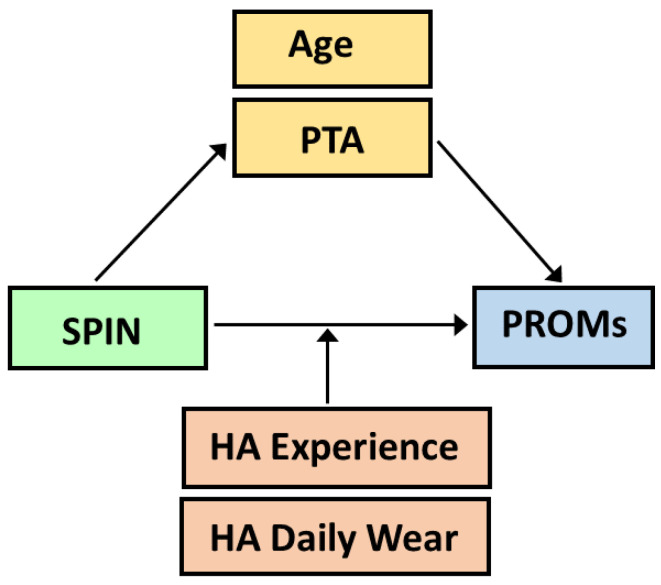
Conceptual path diagram showing the different analyses performed: correlations between speech-in-noise thresholds (SPIN) (aided and unaided SPIN thresholds, aided, and unaided task-related listening effort) and PROMs scores were analysed (EEAS with its EEAS*Quiet* and EEAS*Noise* subscales; 15iSSQ with SSQ*Speech*, SSQ*Spatial* and SSQ*Qualities* subscales). Mediation analysis was used to identify if the SPIN thresholds/PROMs relationships were mediated by hearing thresholds (unaided and aided pure-tone thresholds, PTA), age, via an indirect pathway. HA experience (in years) and HA daily wear (in hours) were used as potential moderators and mediators. Dependent variables are in blue, independent variables (or focus variables) in green, mediator variables in orange and moderators in pink. The same colour scheme has been used throughout the paper.

Moderated mediation analysis allows for identifying how information is shared between two variables, via direct and indirect pathways, and how those relationships can depend on moderator factors. As the aim of the study was to analyse correlations between scores from the SPIN test and PROMs, we built several models that used scores from the PROMs (EEAS and SSQ scores) as the dependent variables and SPIN test scores (HA_SPIN, UA_SPIN, HA_TLE, UA_TLE) as the independent variables or focus variables. The following mediator variables were tested: age, hearing thresholds without and with hearing aids, and total hearing-aid experience. The total duration of hearing-aid experience, in years, and the daily hours of HA wear (HADW) were used as moderators. Figure 1 summarises the different conceptual models tested. These analyses were conducted using the PROCESS macro for R version 4.3.1, authored by Hayes (2022) [67]. For all analyses, confidence intervals (CIs) were derived from 5000 bootstrapped samples and were used to assess the statistical significance of the direct effects, indirect effects, moderation, and coefficients. Confidence intervals not straddling zero were deemed as an index of statistical significance. Significant regions of moderation were identified using Hayes’s bootstrap-based Johnson–Neyman approach. Statistical significance of the moderation is given, together with the percentage of the variance explained by the moderator (r^2^ change). The variables used as moderators were mean-centred, and HA experience, in years, was log transformed prior to statistical analyses.

## 3. Results

### 3.1. Perception Thresholds and PROMs Scores

Table 2 summarises pairwise correlations between the main variables in the study. There was no significant correlation between PROMs and age, but there was a significant increase in listening effort (EEAS) with the total hearing-aid experience (r = 0.45, *p* < 0.01). EEAS increased significantly with increasing hearing loss (r = 0.41, *p* < 0.03), and SSQ decreased with hearing loss (r = −0.51, *p* < 0.003) and with HA hearing thresholds (r = −0.39, *p* < 0.03).

Task-related listening effort threshold (TLE) measured without HA was significantly more correlated to SPIN measured without HA (r = 0.74, *p* < 0.0001), than with HA (r = 0.48, *p* < 0.005), z = 2.22, *p* < 0.02. As expected, TLE significantly correlated with the speech perception thresholds measured in the same corresponding conditions (r = 0.74, *p* < 0.0001 for HA and r = 0.68, *p* < 0.0001 for unaided condition).

EEAS and 15iSSQ shared 60% of the variance (r = −0.77, *p* < 0.0001). Correlations between EEAS and 15iSSQ subscales ranged from r = −0.62, *p* < 0.001, (SSQ*Quality*) to (r = −0.75, *p* < 0.0001 (SSQ*Speech*).

### 3.2. Speech-in-Babble-Noise and 15iSSQ Scores

#### 3.2.1. SSQ*Speech* Scores

In simple pairwise correlations, SSQ*Speech* showed a strong increase with decreasing UA_PTA (r = −0.47, *p* < 0.007) and, to a lesser degree, with HA_PTA (r = −0.38,*p* < 0.03) and HA_SPIN (r = −0.35, *p* < 0.05). A tendency to decrease as HA experience increased was also observed (r = −0.30, *p* < 0.07).

UA_SPIN–SSQ*Speech*

Several simple mediation models were tested to see whether there were any indirect effects of UA_SPIN on SSQ*Speech* through age, HA_PTA, and UA_PTA: no statistically significant indirect effects were obtained except for a tendency for small mediation by HA_PTA. However, a strong moderating influence was observed on a direct path between unaided SPIN tests and SSQ*Speech*.

**Figure 2:**
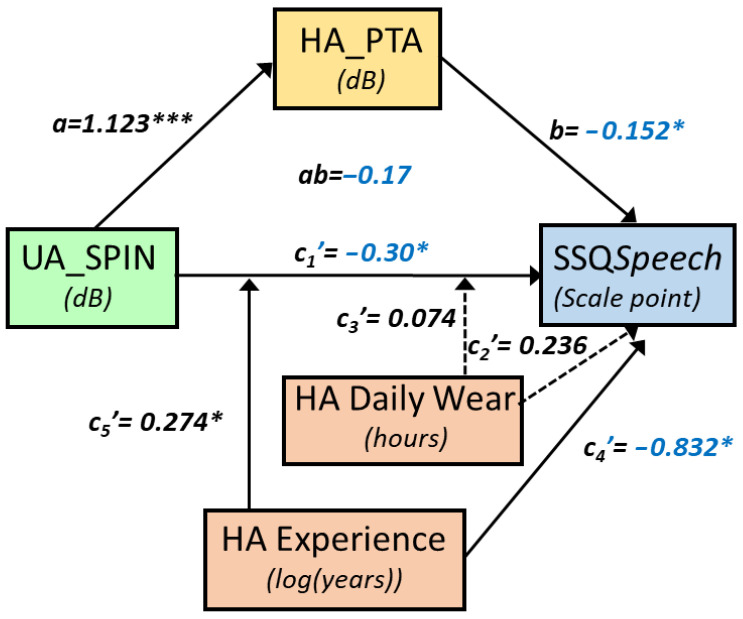
Conceptual path diagram showing the relationship between the unaided SPIN threshold (UA_SPIN) and SSQ*Speech*. Continuous arrows show statistically significant links (*p* < 0.05), whereas dotted arrows symbolise nonsignificant paths. For readability, negative coefficients are shown in blue. The direct path was under moderation by both HA experience and hearing-aid daily wear (HADW) in hours. The coefficients shown are for mean-centred HA experience and HADW. Stars indicate statistical significance, with * for *p* < 0.05, *** for *p* < 0.001.

**Figure 3:**
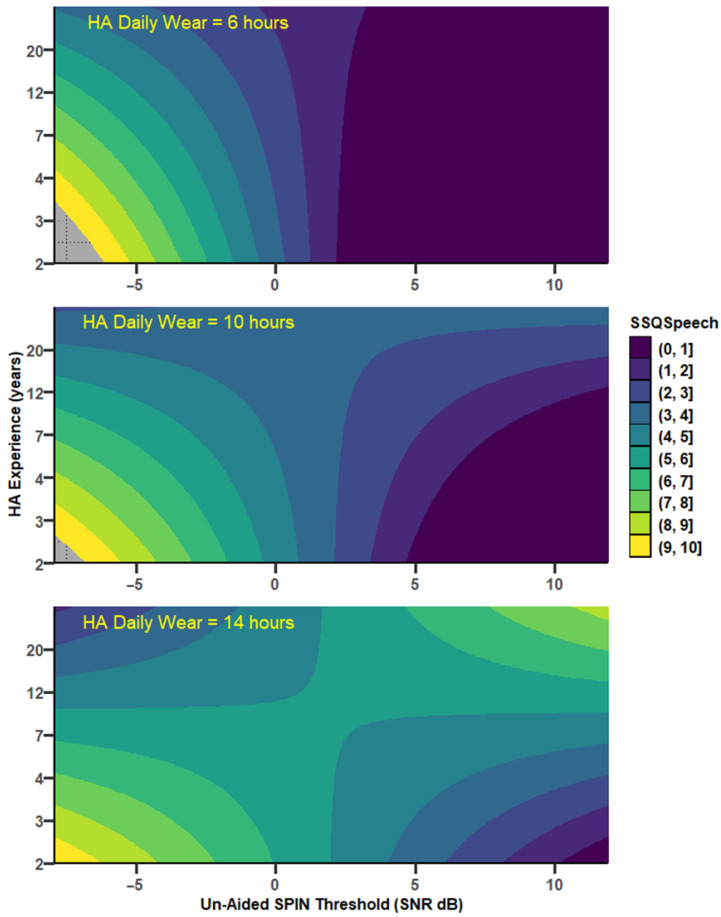
Predicted SSQ*Speech* scores from the model depicted in Figure 2. SSQ*Speech* scores vary between 0 (dark colours, worse hearing abilities) and 10 (light colours, good hearing abilities), as a function of the hearing threshold in cocktail party noise (UA_SPIN) and HA experience (years). The strong correlation between UA_SPIN and SSQ*Speech*, visible for low HA experience values, disappear for long HA experiences. Each panel represents a different value of HADW, the second moderator: the relationship between UA_SPIN and SSQ*Speech* is stronger for low values of HADW.

SSQ*Speech* increased significantly with the unaided SPIN threshold in a mediated and moderated model (r = 0.75, MSE = 2.65, *p* = 0.0012, Figure 2). There was a tendency for a decrease in SSQ*Speech* as UA_SPIN increased via HA_PTA. Specifically, HA_PTA increased as UA_SPIN increased (a = 1.123, *p* < 0.0001, 95%CI [0.77, 1.48]), and SSQ*Speech* decreased as HA_PTA increased (b = −0.15, *p* = 0.05, 95%CI [−0.30, 0.00], with ab = −0.17, 95%CI [−0.35, 0.02]. As confidence intervals straddle 0, this indirect path is not considered as statistically significant. However, the direct path was strongly moderated by both HA experience, which uniquely accounted for 7.7% of the variance change (*p* < 0.05), and HADW, which uniquely accounted for a nonsignificant 2.5% of the variance. However, the joint test of interaction showed that 31.2% of the variance (*p* = 0.0012) was accounted for when the UA_SPIN effect was allowed to vary as a function of both HA experience and HADW. Through the direct path, there was a significant decrease of SSQ*Speech* by 0.30 point, (se = 0.14, *p* = 0.043) as UA_SPIN increased by 1 dB for mean HA experience and HADW. For those mean values, SSQ*Speech* decreased significantly as HA experience increased (*p* = 0.03) as well. The effect of UA_SPIN on SSQ*Speech* was present for HA experiences below 2.6 years and for HADW below 12 h daily. For instance, for 1.63 years of HA experience and 8.3 h of HADW, SSQ*Speech* decreased by 0.75 points (se = 0.24, *p* < 0.005) as UA_SPIN increased by 1 dB. Figure 3 shows the SSQ*Speech* predicted values from the model (coloured, light shades indicating good hearing capabilities), as a function of the unaided SPIN threshold and HA experience, and for different levels of HADW.

UA_TLE–SSQ*Speech*

**Figure 4:**
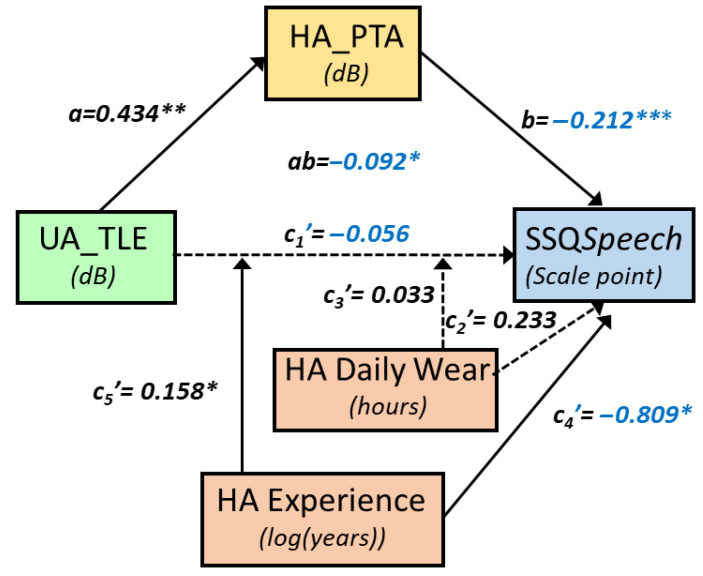
Conceptual path diagram showing the relationship between unaided task-related listening effort (UA_TLE), in speech-in-noise, and SSQspeech scores. Continuous arrows show statistically significant links, whereas dotted arrows symbolise non significant paths. For readability, negative coefficients are shown in blue. The direct path was under moderation by both HA experience and hearing-aid daily wear in hours and was not statistically significant for mean values of the moderators (HA experience and HADW, which are mean-centred). However, the direct path was statistically significant for low values of both HA experience and HADW. Stars indicate statistical significance, with * for *p* < 0.05, ** for *p* < 0.01 and *** for *p* < 0.001.

UA_TLE was correlated with SSQ*Speech*, according to a similar model as UA_SPIN (r = 0.75, MSE = 2.65, *p* = 0.0012, Figure 4).

**Figure 5:**
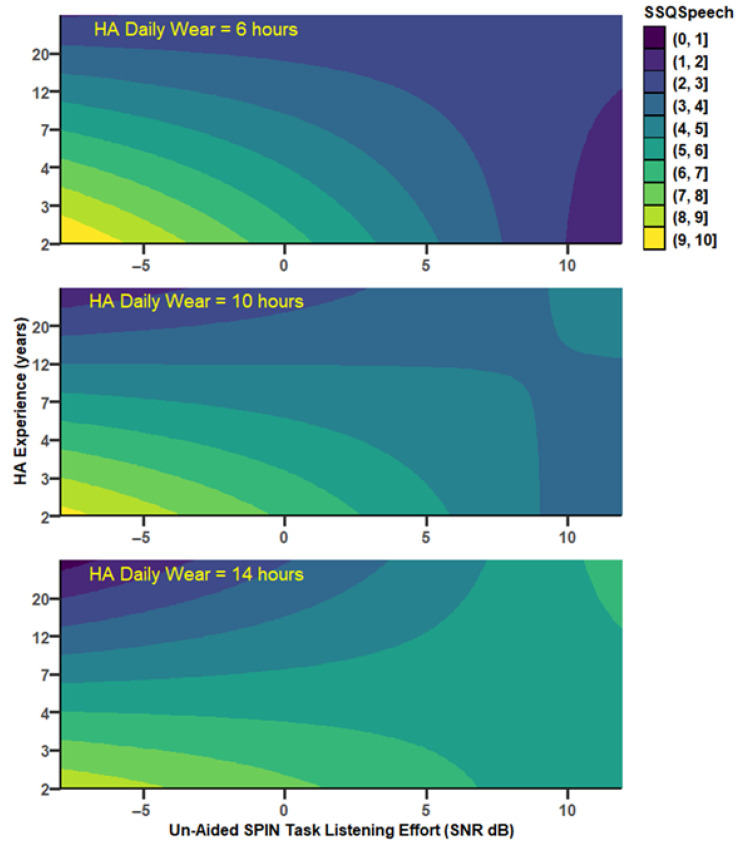
Predicted SSQ*Speech* scores from the model depicted in Figure 4. SSQ*Speech* scores vary between 0 (dark colours, worse hearing abilities) and 10 (light colours, good hearing abilities), as a function of the threshold for task-related listening effort in cocktail party noise (UA_TLE) and HA experience (years). The strong correlation between UA_TLE and SSQ*Speech*, visible for low HA experience values, disappear for longer HA experience. Each panel represents a different value of HADW, the second moderator.

Specifically, SSQ*Speech* decreased by 0.09 points (ab = −0.092, se = 0.05, 95%CI [−0.196, −0.001]) as UA_TLE increased by 1 dB, through HA_PTA (a = 0.434, se = 0.14, *p* = 0.0041, 95%CI [0.149, 0.719] and b = −0.212, se = 0.06, *p* = 0.0008, 95%CI [−0.327, −0.098]). The direct path was strongly moderated by HA experience, which accounted uniquely for 9.6% of the variance (*p* < 0.03), and by HADW, which accounted for a nonsignificant 2.6% of the variance. The joint test of interaction showed that both moderators together accounted for 30.6% of the variance (*p* = 0.0013). For HA experience below 2.2 years and HADW below 9.5 h, SSQ*Speech* decreased significantly as UA_TLE increased. For instance, for 2 years of HA experience and 9 h per day of HADW, SSQ*Speech* decreased by 0.23 points (se = 0.09, *p* < 0.02) as UA_TLE increased by 1 dB (Figure 5).

HA_SPIN–SSQ*Speech*

**Figure 6:**
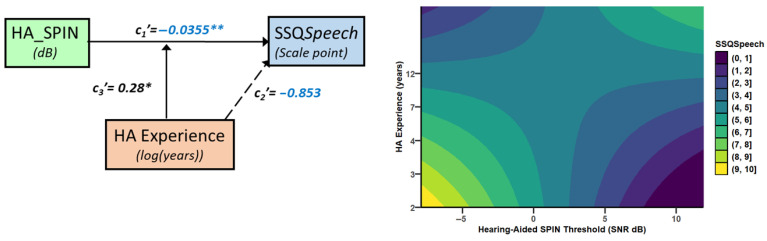
The left panel shows a simple moderated model of the relationship between the hearing-aided SPIN threshold and SSQ*Speech*. The right panel shows the predicted values of SSQ*Speech* by the model, as a function of HA_SPIN and HA experience, with the moderation by HADW. Stars indicate statistical significance, with * for *p* < 0.05, ** for *p* < 0.01.

A similar model built with HA_SPIN as the focus variable violated the assumption of independence between the focus variable and mediator (HA_PTA). Therefore, we performed a simple moderated model. In this model (r = 0.57, MSE = 3.64, *p* = 0.01, Figure 6), SSQ*Speech* increased significantly by 0.36 points (se = 0.12, *p* < 0.008) for the mean HA experience when HA_SPIN decreased by 1 dB. This relationship was significantly moderated by HA experience (r^2^ change = 0.15, *p* = 0.02); significant increase in SSQ*Speech* with decreasing HA_SPIN occurred only for less than 6 years of HA experience. For 2.6 years of HA experience (median of the HA experience), the rate of SSQ*Speech* increase was 0.453 point per dB of HA_SPIN decrease (se = 0.15, t = −3, *p* = 0.0054). Contrary to the model with UA_SPIN, there was no significant moderation by HADW (Figure 6).

HA_TLE–SSQ*Speech*

**Figure 7:**
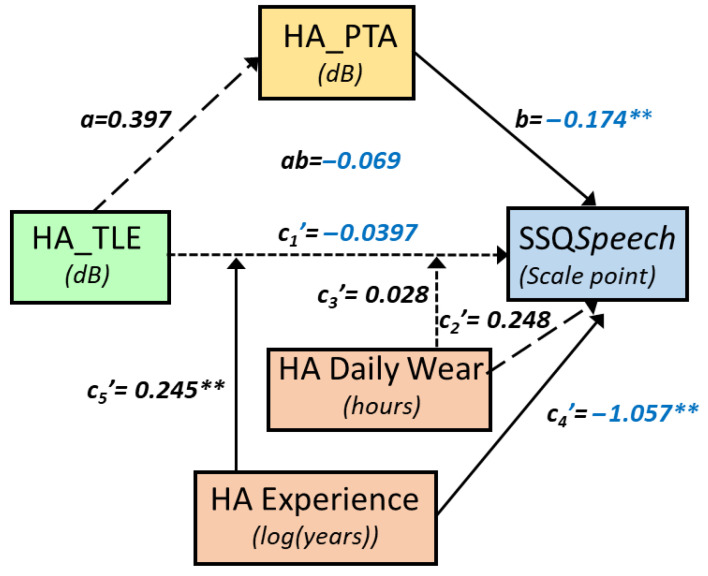
Conceptual diagram showing the relationship of SSQSpeech with the hearing-aided task-related listening-effort threshold (HA_TLE). The small indirect pathway via the hearing-aided threshold (HA_PTA) is not significant (*p* < 0.10). The direct effect is strongly moderated by both HA experience and HADW, and is significant for low values of HA experience and low values of HADW. HADW and HA experience have been mean-centred. Stars indicate statistical significance, with ** for *p* < 0.01.

**Figure 8:**
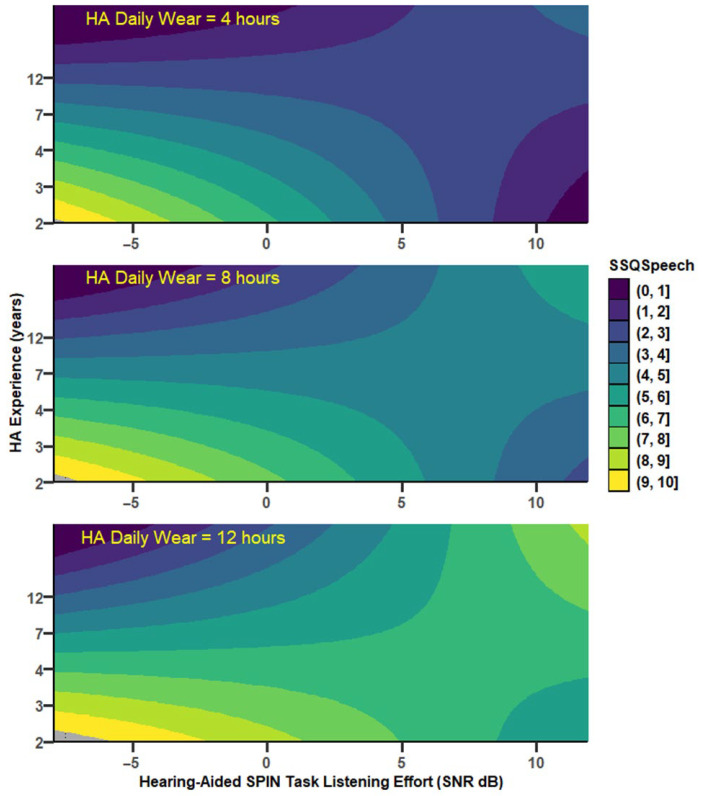
Predicted SSQ*Speech* scores from the model depicted in Figure 7. SSQ*Speech* scores vary between 0 (dark colours, worse hearing abilities) and 10 (light colours, good hearing abilities), as a function of the threshold for hearing-aided task-related listening-effort in cocktail party noise (HA_TLE) and HA experience (years). The strong correlation between HA_TLE and SSQ*Speech* visible for low HA experience values disappear for larger values of HADW. Each panel represents a different value of HADW, the second moderator.

The model built with HA_TLE as the focus variable (r = 0.75, MSE = 2.68, *p* = 0.0013), shows a nonsignificant indirect path through HA_PTA and a strong moderation by both HA experience and HADW on the direct path (Figure 7). HA experience accounted uniquely for 14.9% of the variance (*p* < 0.008) and HADW accounted for a nonsignificant 3.2% of the variance, but both moderators together accounted for 31.45% of the variance (*p* = 0.0012). For HA experience below 2.2 years and HADW below 8.4 h, SSQ*Speech* decreased significantly as HA_TLE increased. For instance, for 2 years of HA experience and 8 h per day of HA_Daily wear, SSQ*Speech* decreased by 0.28 points (se = 0.094, *p* < 0.007) as HA_TLE increased by 1 dB (Figure 8).

#### 3.2.2. SSQ*Quality*

Pairwise correlations (Table 2) showed that SSQ*Quality* increased significantly with decreasing HA_SPIN (r = −0.50, *p* < 0.004), HA_TLE (r = −0.50, *p* < 0.004) and HA_PTA (r = −0.38, *p* < 0.04). Similarly, there was a significant SSQ*Quality* decrease with increasing unaided thresholds: r = −0.46, *p* < 0.01 for UA_TLE and r = −0.44 (*p* < 0.012) for UA_PTA, but no statistical significance with UA_SPIN (r = −0.33, *p* < 0.07).

No statistically significant evidence of either mediation or moderation was observed for the relationship between SSQ*Quality* and HA_SPIN, UA_SPIN, or HA_TLE.

**Figure 9:**
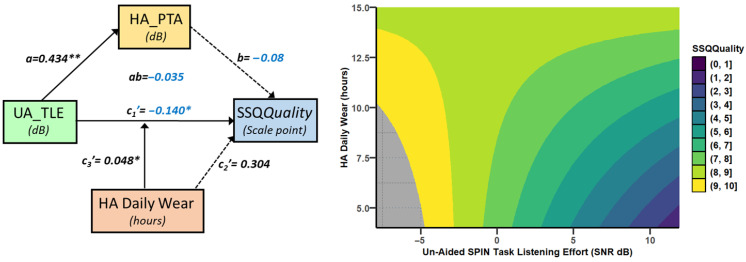
The left panel shows the model of the relationship between unaided SPIN task-related listening effort SPIN (UA_TLE) and SSQ*Quality*. The right panel shows the predicted values of SSQ*Quality* by the model as a function of UA_TLE and HADW. There was no significant moderation. Stars indicate statistical significance, with * for *p* < 0.05, ** for *p* < 0.01.

The relationship between UA_TLE and SSQ*Quality* was moderated by HADW (r^2^ change = 0.11, *p* < 0.04), in a model mediated by HA_PTA (r = 0.60, MSE = 2.57, *p* < 0.02). The indirect pathway through HA_PTA was not significant (ab = 0.035, se = 0.034, 95%CI [−0.106, 0.023]). Through the direct path, SSQ*Quality* increased by 0.14 points (se = 0.052, *p* < 0.02) for a decrease of 1 dB of UA_LTE, for the mean HADW (11.4 h). The relationship between SSQ*Quality* and UA_LTE was significant only for HADW below 12.2 h per day (Figure 9).

#### 3.2.3. SSQ*Spatial*

Weak or absent significant correlations were obtained between SSQ*Spatial* and the focus variables (see Table 2 for details).

### 3.3. Speech-in-Babble-Noise Perception and Daily Listening Effort (EEAS)

**Figure 10:**
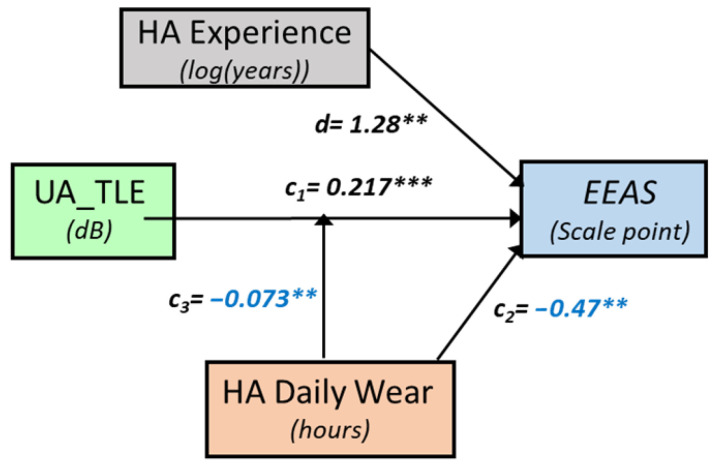
Conceptual diagram showing the relationship between unaided SPIN task-related listening effort (UA_TLE) and daily listening effort (EEAS). HA daily wear is a significant moderator. For readability, negative coefficients are shown in blue. Stars indicate statistical significance (** *p* < 0.01; *** *p* < 0.001). The coefficients presented are for a mean-centred HA daily wear.

Correlations between EEAS and SPIN tests were not mediated significantly by any of the mediator variables (PTA, age, HA experience, nor HADW). However, EEAS usually increased significantly as both UA SPIN thresholds and HA experience increased, with a moderation by HADW (Figure 10): the relationship between EEAS and predictors were stronger for HADW below 13 h. EEAS depended as well, but to a lesser degree, on aided SPIN and HA experience, without any significant moderation by HADW. The EEAS*Noise* subscale behaved similarly to EEAS. The EEAS*Quiet* subscale showed significant increase as both UA_SPIN and HA experience increased, but, as well, a significant decrease as HADW increased, with a strong moderation by HADW (more than 13% variance explained by the interaction between UA SPIN tests and HADW). Overall, the correlations between EEAS and unaided SPIN were stronger than between EEAS and aided SPIN. All the different models with coefficients are available in Supplemental Table S2).

#### 3.3.1. Daily Listening Effort and Unaided SPIN

**Figure 11:**
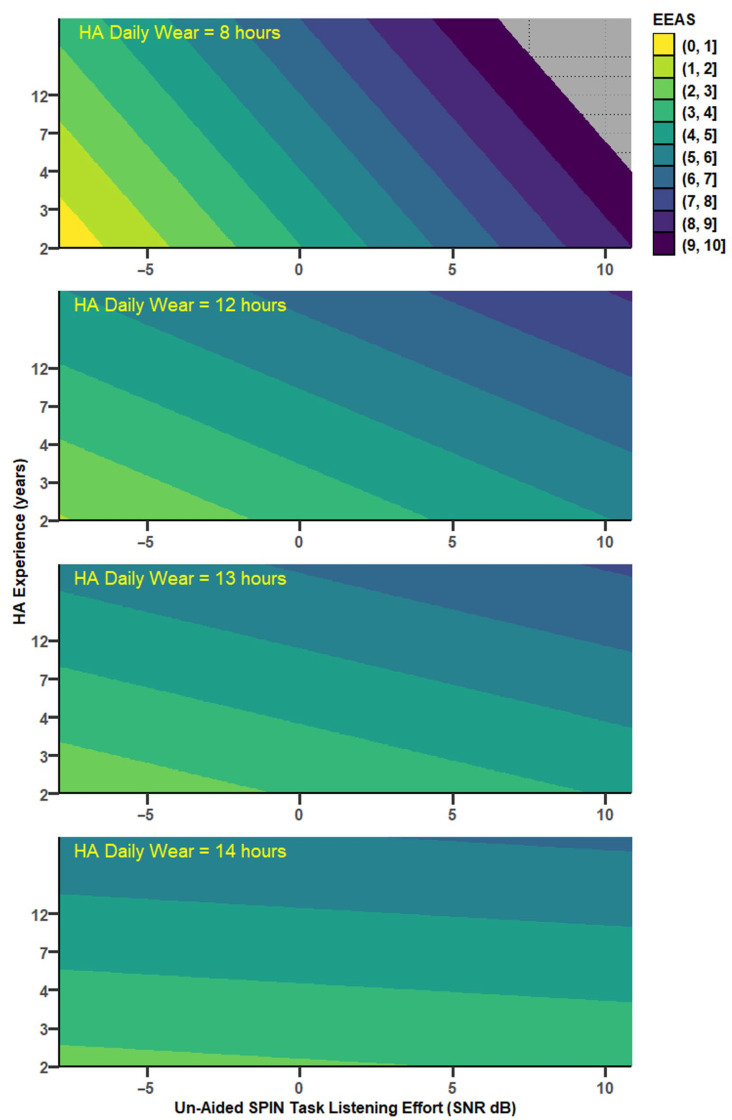
Predicted values of daily listening effort (EEAS) as a function of HA experience (in years) and unaided SPIN task-related listening effort, for different levels of HA daily wear, which was a significant moderator of the model, depicted in Figure 10. Dark colours depict maximum daily listening effort whereas light colours depict minimum listening effort (and better hearing).

EEAS depended significantly on UA_SPIN, HA experience, and HADW (r = 0.67, MSE = 3.36, *p* = 0.0025), with a significant moderation by HADW (r^2^ change = 0.16, *p* = 0.01), for values of HADW below 12.8 h. A similar model was obtained with UA_TLE (Figure 10); EEAS depended significantly on UA_TLE, HA experience and HADW (r = 0.74, MSE = 2.73, *p* = 0.0002), with a significant moderation by HADW (r^2^ change = 0.18, *p* = 0.0029), for values of HADW below 13 h (Figure 11).

EEAS*Noise* behaved in a similar manner as EEAS, with a strong dependency on HA experience, increasing with HA experience (*p* < 0.007) but no significant moderation by HADW (*p* = 0.074), and a tendency to increase with UA_SPIN (*p* = 0.05). However, EEA*Noise* increased significantly when both UA_LTE and HA experience increased (r = 0.68, MSE = 4.28, *p* = 0.0016), and this relationship was significantly moderated by HADW (r^2^ change = 0.095, *p* < 0.04), with significant relationship for HADW below 13 h.

EEAS*Quiet* was significantly dependent on all variables entered into the model, with UA_SPIN (r = 0.61, MSE = 3.59, *p* = 0.01) increasing as both UA_SPIN and HA experience increased, and decreasing with increasing HADW, with a significant interaction with HADW (r^2^ change = 0.13, *p* < 0.03) for HADW below 13 h. When UA_LTE was used, EEAS*Quiet* showed a strong dependency on UA_LTE (*p* = 0.003) and on the interaction between UA_LTE and HADW (r^2^ change = 0.154, *p* < 0.01), but neither HA experience nor HADW reached statistical significance (*p* < 0.10).

#### 3.3.2. Daily Listening Effort (EEAS) and Aided SPIN

EEAS increased significantly with increasing HA_SPIN and HA experience (r = 0.63, MSE = 3.7, *p* < 0.008), without any significant moderation by HADW. EEAS increased significantly with both HA_TLE and HA experience (r = 0.64, MSE = 3.5, *p* < 0.005), without any evidence of significant moderation by either HA experience or HADW.

EEAS*Noise* increased significantly with HA experience, but not with HA_TLE nor with HA_SPIN, and no significant moderation was obtained by HADW.

EEAS*Quiet* increased significantly with increasing HA_SPIN (and with HA_TLE), but did not depend significantly on HA experience nor on HADW.

## 4. Discussion

This study was aimed at analysing relationships between speech-in-babble-noise perception thresholds and two outcome auditory questionnaires, the 15iSSQ short form of the Speech Spatial and Qualities of Hearing Scale, and the Extended Listening Effort Assessment Scale, in hearing-aided patients.

### 4.1. SSQSpeech Scores Increase with Both Unaided and Aided SPIN Thresholds

We obtained a significant decrease of SSQ scores (and increase of EEAS scores), including their subscales, as hearing-aided speech-in-babble-noise thresholds increased. Although simple pairwise correlation coefficients appeared greater between PROMs and HA SPIN than between PROMs and UA SPIN, no significant differences between correlation coefficients were obtained. As all patients were experienced HA users and were specifically instructed to complete the PROMs as if they were wearing their hearing aids in the situations depicted, we would have expected a closer relationship between HA_SPIN and PROMs scores. The fact that the patients were using their usual hearing aids during the SPIN tests, and that a highly reproducible speech test was used, might explain the discrepancy between the present study, where strong SSQ/SPIN relationships were obtained, and the lack of correlation between SSQ and speech-in-noise tests observed in a large hearing-aided population [22]. We used a 16-voice babble noise, closer to the situations depicted in the PROMs, but a closed-set test, less prone to top-down contextual influences than open-set tests. The better reproducibility of such closed-set tests could favour the correlation with PROM scores, even though such tests are less ecologically valid than open-set sentence-based tests. Indeed, previous correlations between the SSQ and SPIN have been obtained with either closed-set tests such as the digit triplet test [42,49], or the consonant discrimination test [40], with no correlation obtained, in the same populations when sentence-based tests were used [40,49]. In 98 people with hearing impairment, most of whom were not hearing-aid wearers, a SSQ global score showed dependence on a global cognitive score, a hearing loss score, and on an environmental sound identification test in Gaussian noise [68]. A significant correlation was observed between this global SSQ score and global-aided speech understanding in noise score (r = 0.26) [68]. In the present study, correlations between unaided PTA or SPIN and PROMs were not statistically different from the same correlations with aided PTA or SPIN. This can be ascribed to the fact that signal intensity was adapted to each patient, so that their speech perception in quiet was above 95%, partially compensating for hearing loss, as the aim was to assess specifically speech-in-babble-noise deficit for equivalent and good speech in quiet scores. This would explain that more than 50% of the variance was shared between HA_SPIN and UA_SPIN, and, also, between HA_TLE and UA_TLE. Further, we obtained a close relationship between task-related listening effort and SPIN in the same condition, with or without hearing aids.

### 4.2. Task-Related Listening Effort Threshold (TLE) and PROMs Scores

The task-related listening effort threshold (TLE) increased significantly with the listening effort in daily life, as assessed by the EEAS. As both measures assess listening effort, such correlations were expected, although we would have expected a closer relationship between EEAS and listening effort assessed during the aided speech-in-noise task, as both related to measures performed with hearing aids. Contrary to the EEAS, 15iSSQ was weakly correlated to the TLE, and the correlation was driven by 15iSSQ*quality*.

Indeed, an equivalent strong correlation was obtained between task-related listening effort and 15iSSQ*Qualities*, which encompass three of the four items that consider “*identification of sounds and objects*” in the full SSQ*Quality*. Most studies relating SSQ with speech-in-noise scores have either used specifically the SSQ*Speech* [20,23,40,43] or the SSQ12 short form [41], which represent the speech subscale more than other subscales, so that the literature dealing with SSQ*Quality* is scarce and devoted to normal-hearing people of various ages. In their normal-hearing multi-age population, Stenbäck et al. [22] report decreasing SSQ*Quality* scores as the HINT threshold increases (but to a lesser degree than SSQ*Speech*), and similar links between the different SSQ subscales and digit triplet test scores in babble noise are reported in a group of 67 young people with normal hearing [42]. It is worth noting that the items in 15iSSQ*Quality* used in the present study relate to “*identifying people by voice*”, “*distinguishing familiar music, different sounds”*, and “*clarity of everyday sounds*”—all are abilities that would specifically be helpful in distinguishing the signal from 16-voice babble noise, especially as both signal and noise were not spatially separated. As the ability to distinguish voices and sounds decreased (15iSSQ*Quality*), the listening effort assessed in the speech-in-babble-noise task increased. It is possible that, in a speech-shaped noise, such correlations with 15iSSQ*Quality* would not be obtained. Interestingly, in the normal hearing population of various age of Stenbäck et al. (2023) [22], correlation between SSQ*Quality* and speech-in-noise tests reached statistical significance with the HINT test score (in speech-shaped noise) and with the Hagerman sentence test with the four-talker babble noise (but not with the same test in the speech-shaped noise). However, the full SSQ*Quality* encompasses items that load more on the SSQ*Speech* subscale than on the quality subscale. (items 3, 14, 17, 18, and 19 of the full SSQ*Quality* did not load well on the qualities subscale, according to two exploratory factor analyses on an English SSQ version [34] and on the corresponding French SSQ version [28]). For that reason, these problematic items were eliminated from the 15iSSQ*Quality* subscale [38]. 15iSSQ*Quality* also retained items pertaining to “*naturalness of music*” and “*clarity of everyday sounds*”, which are more relevant to hearing-aided people than to unaided people, as hearing-aid related distortions can specifically affect those two items. This could explain why 15iSSQ*Quality* shows a stronger relationship with HA_SPIN than with unaided SPIN, and drives the correlation between 15iSSQ and HA_SPIN. Contrary to the more complex relationship between the 15iSSQ*Speech* and SPIN variables, 15iSSQ*Quality* relationship with SPIN is not mediated, nor is it moderated by the total experience with hearing aids. However, the relationship between UA_TLE and SSQ*Quality* is moderated by HA daily usage and becomes significantly stronger as daily usage falls below 12 daily hours. It seems that the patients with relatively low daily HA use tend to answer, at least partly, the 15iSSQ*Quality* items as if they were not wearing their hearing aids.

Contrary to both 15iSSQ*Speech* and 15iSSQ*Quality*, the relationship between the SSQ*Spatial* and SPIN remained weak or nonsignificant, and was not moderated by any of the variables. As all patients presented symmetrical hearing loss, and as the signal and noise were not spatially separated during the SPIN tests, it is likely that those conditions did not favour a potential relationship between 15iSSQ*Spatial* and Speech in noise thresholds, as 15iSSQ*Spatial* pertains to sound localisation and is strongly predicted by hearing asymmetry [52].

### 4.3. A Moderating Influence by HA Experience and Daily Usage

The moderating influence by HA usage and experience is of major importance for both the 15iSSQ*Speech* and EEAS results, and is one of the major results of this study because it could explain, at least partly, the relatively weak and unstable relationship between PROMs and speech-in-noise tests across different studies. Indeed, as all of our HA wearers had more than 1.5 years of HA experience (mean of 5.6 years), with a mean daily usage of more than 11 h, we would expect a closer relationship between the PROMs and their HA SPIN than with their unaided SPIN, in the models including mediation and moderation. This was not the case, with a stronger relationship with both the unaided SPIN and listening-effort thresholds, when compared to the hearing-aided thresholds. Although our patients were specifically instructed to complete the PROMs as they wear their hearing aids in the situations depicted in the PROMs, it seems that their self-assessment reflected, at least partly, their experience without hearing aids. Indeed, relationships between the unaided SPIN and PROMs was stronger for low experience with HA and relatively low daily usage, with cutoff values around 3 years of HA experience and 8 h of HADW for 15iSSQ*Speech* and EEAS. This could be interpreted as a much longer period of time than previously thought, needed for the patients to really self-assess their hearing abilities with HA, as opposed to without HA. HA acclimatisation has been described within the first weeks following HA fitting [69,70,71,72] and might continue to a lesser degree within the first 6 months. The entire population of the present study has been fitted for more than 18 months, which is well outside the period of HA acclimatisation. Therefore, it is likely that the evolution of the SPIN–PROMs relationship with HA use and experience is due to an evolution in how patients self-assess their abilities in the PROMs, during the first few years after initial HA fittings. Indeed, process analysis showed a strong synergistic moderating influence of both HADW and HA experience, in the relationship between 15iSSQ*Speech* and UA_SPIN: for low values of HA experience and HADW, 15iSSQ*Speech* can decrease by 0.75 points for each dB increase of UA_SPIN. These moderating influences could account for more than 30% of the variance in both UA_SPIN and UA_TLE, with a stronger influence of HA experience than HADW. According to the model, for a given HADW and UA_SPIN (or UA_TLE), there is a decrease in SSQ*Speech* scores as HA experience increases, although this relationship does not reach statistical significance in a simple correlation between 15iSSQ*Speech* and HA experience when no other variable is taken into account or partialled out. This strong moderation by both HA experience and HADW on the relationship between SSQ*Speech* and the speech-in-babble-noise tests could explain discrepancies obtained in the international literature regarding the PROMs and hearing-in-noise tests relationships. In addition, part of the relatively stronger 15iSSQ*Speech*–SPIN relationship obtained here could be attributed to the better sensitivity of 15iSSQ*Speech* towards hearing loss, when compared to the full SSQ*Speech*. Indeed, for a group of hearing-impaired non-hearing-aided patients and a group of hearing-aid wearers, the receiver-operating characteristic curves showed an area under the curve significantly greater for 15iSSQ*Speech* than SSQ*Speech* [38].

EEAS scores have already been shown to strongly depend on HA experience, with an increase in daily listening effort as HA experience increases beyond 2 years of HA experience, with no mediation by either hearing loss or age [59]. The present study confirms the strong dependence of EEAS, especially EEAS*Noise*, on HA experience: EEAS increased significantly as both HA experience and UA_TLE increased and HADW decreased (e.g., Figure 10). Furthermore, the relationship between EEAS and UA_SPIN (and UA_LTE) was significantly moderated by HADW: low HADW values were associated with stronger relationships between UA_TLE (and UA_SPIN) and EEAS. When HA tests were involved, EEAS increased significantly with increases in both HA_SPIN and HA experience, but those relationships were not moderated by either HA experience or HADW. In addition, it appears that, similar to EEAS, 15iSSQ*Speech* and 15iSSQ*Quality* seem to deteriorate with increasing HA experience for a given SPIN threshold.

### 4.4. Limitations of the Study

In the same way as relationships between PROMs and unaided tests grow stronger for low HA experience and low HADW, we would expect that relationships between PROMs and aided tests would grow stronger with HA experience. Unfortunately, as most of our HA wearers have HA experience of less than 5 years, the present study does not show this potential counterpart moderating influence. Indeed, the limited range of HA experience and number of patients did not allow us to further refine the limits in HA experience and HADW for which relationships between PROMs and SPIN tests are modified. The average HA daily wear by our patients was more than 11 h daily, with no patients below 5 h, which restricts the applicability of our results to patients that are extensive HA users. Results could also depend on the type and trademark of the HAs and gain provided per frequency, but the sample studied did not allow for assessing detailed influence of such variables.

To increase reproducibility, we chose a closed-set test of speech perception, with collocated signal and noise to avoid variability linked to spatial cues, which can be differentially accounted for by various HAs. The closed-set tests decrease some of the variability due to contextual influences on more complex speech material, such as sentences, but, conversely, are further from typical patient experiences in noisy environments. Lastly, the mean age (77.5) and the wide age range (SD = 12.3) in our population is slightly greater than large-scale studies (70.5 years (SD = 11.8) for 1751 HA wearers from Norway [8], or 72.9 years (SD = 9.9) for 581 American HA wearers [73]) since we did not use a fixed upper age limit. However, our results reflect random sample differences and the typical age range for experienced HA wearers in a hearing-aid dispensary.

## 5. Conclusions

Despite long-term development of several auditory patient-reported outcome measures, the relationships between these PROMs and speech-perception-in-noise scores has yielded several contradictory results and has been little explored in patients with conventional hearing aids. The present study aimed to analyse the interrelations among (1) self-report auditory scales (15-item short-form of the Speech Spatial and Qualities of Hearing Scale (15iSSQ) and the Extended Listening Effort Assessment Scale (EEAS)); (2) speech perception in cocktail-party noise, measured with and without HAs; and (3) self-assessment of the listening effort perceived during the speech-in-noise perception task.

Results showed a strong relationship between PROMs (EEAS and 15iSSQ, especially 15iSSQ*Speech* and 15iSSQ*Quality*) and SPIN assessed with and without HAs in HA wearers. Most importantly, it shows a major moderating factor by both HA experience and HADW, even for a population that is well outside the time usually described as the acclimatisation period, and that wear their HA for more than 11 h daily on average. Low HA experience (but still over 18 months) and low HADW are associated with much stronger relationships between SSQ scores and SPIN, especially when those are obtained without HAs.

Both SSQ and EEAS showed a relationship with SPIN; although sharing similar daily communication situations, SSQ and EEAS indicate different dependencies, with EEAS being very strongly related to HA experience.

This study emphasises the importance of taking HA experience and daily wear into account when analysing correlations between self-report difficulties in daily life and laboratory tests in noise, in experienced HA wearers.

## Figures and Tables

**Table 1:** Patients characteristics (n = 32 patients), thresholds, and PROMs scores (15iSSQ and subscales, EEAS and subscales). PTA—pure-tone audiometry, SPIN—speech in cocktail-party noise threshold, SNR—signal to noise ratio. Mean and standard deviations (SD).

Variables	Mean	SD
Age (years)	77.53	12.31
Unaided PTA (best ear) average 0.5, 1, 2, and 4 kHz thresholds (dB HL)	50.20	12.59
Aided PTA (best Ear) average 0.5, 1, 2, and 4 kHz thresholds (dB HL)	28.67	6.33
Unaided hearing asymmetry (dB HL)	5.82	4.39
Aided PTA asymmetry (dB HL)	2.66	2.66
Experience with hearing aids (years)	5.60	7.02
Daily wear of hearing aids (hours)	11.35	2.94
Unaided SPIN threshold (dB SNR)	−1.18	4.31
Hearing-aided SPIN threshold (dB SNR)	−1.11	3.48
Unaided SPIN task-related listening effort threshold (dB SNR)	4.02	7.21
Hearing-aided SPIN task-related listening effort threshold (dB SNR)	3.16	5.29
15iSSQ score (from 0 (cannot do) to 10 (do perfectly))	6.63	1.85
SSQ speech (from 0 to 10)	5.61	2.21
SSQ spatial (from 0 to 10)	6.45	2.30
SSQ quality (from 0 to 10)	7.84	1.86
EEAS (from 0 (no effort) to 10 (maximum effort))	4.70	2.29
EEASquiet (from 0 to 10)	2.63	2.24
EEASnoise (from 0 to 10)	5.74	2.64

**Table 2:** Pairwise correlations (Pearson correlation coefficients) between the different variables of interest, collected in 32 patients: hearing-ability scores (15iSSQ) (with the three subscales: speech, spatial, and qualities of hearing), daily listening effort scores (EEAS), in quiet (EEAS*Quiet*) and in noise (EEAS*Noise*). Age is represented in years; HA Exp. represents the total experience with hearing aids (log transformed); HADW represents daily wear of hearing aids, in hours; UA_PTA: unaided pure-tone threshold averaged across 500 Hz, 1, 2, and 4 kHz on the better ear; HA_PTA: hearing-aided pure-tone threshold averaged across 500 Hz, 1, 2, and 4 kHz on the better ear; UA_SPIN is the unaided speech-in-babble-noise threshold in dB SNR; HA_SPIN is the hearing-aided speech-in-babble-noise threshold in dB SNR, whereas UA_TLE and HA_TLE represent the threshold (in dB SNR) for a listening effort quantified as 5/10, in the unaided and aided SPIN test, respectively. Significant correlation coefficients are noted in bold (*p* < 0.05), with pale grey background for *p* < 0.01, and darker grey background for *p* < 0.001.

	15iSSQ	SSQ *Speech*	SSQ *Spatial*	SSQ *Qual*	EEAS	EEAS *Quiet*	EEAS *Noise*	Age	HA Exp.	HADW	UA PTA	HA PTA	UA SPIN	HA SPIN	UA TLE
**15iSSQ**	**1.00**														
**SSQ*Speech***	**0.83**	**1.00**							***p*** ** < 0.05**						
**SSQ*Spatial***	**0.92**	**0.62**	**1.00**						***p*** ** < 0.01**						
**SSQ*Quality***	**0.86**	**0.52**	**0.77**	**1.00**					***p*** ** < 0.001**						
**EEAS**	**−0.77**	**−0.75**	**−0.64**	**−0.62**	**1.00**										
**EEAS*Quiet***	**−0.74**	**−0.67**	**−0.66**	**−0.58**	**0.80**	**1.00**									
**EEAS*Noise***	**−0.68**	**−0.64**	**−0.57**	**−0.57**	**0.95**	**0.63**	**1.00**								
**Age**	0.02	−0.21	0.20	0.04	−0.03	0.05	−0.11	**1.00**							
**HA Exp.**	−0.29	−0.32	−0.25	−0.19	**0.45**	0.33	**0.46**	**−0.38**	**1.00**						
**HADW**	−0.03	−0.04	−0.03	0.01	0.04	−0.01	0.12	−0.08	0.18	**1.00**					
**UA_PTA**	**−0.51**	**−0.47**	**−0.42**	**−0.44**	**0.41**	**0.39**	**0.33**	0.14	0.29	0.07	**1.00**				
**HA_PTA**	**−0.39**	**−0.38**	−0.27	**−0.38**	0.27	0.26	0.20	**0.48**	0.05	0.04	**0.65**	**1.00**			
**UA_ SPIN**	−0.30	−0.28	−0.18	**−0.33**	**0.36**	**0.40**	**0.31**	**0.46**	0.17	0.20	**0.46**	**0.76**	**1.00**		
**HA_SPIN**	**−0.47**	**−0.35**	**−0.40**	**−0.50**	**0.44**	**0.49**	**0.37**	**0.33**	0.28	0.16	**0.57**	**0.61**	**0.72**	**1.00**	
**UA_TLE**	−0.29	−0.14	−0.21	**−0.45**	**0.47**	**0.49**	**0.47**	0.14	0.20	0.22	**0.35**	**0.49**	**0.74**	**0.52**	**1.00**
**HA_TLE**	**−0.36**	−0.19	−0.29	**−0.50**	**0.48**	**0.45**	**0.48**	0.04	0.29	0.24	**0.40**	**0.33**	**0.48**	**0.68**	**0.73**

## Data Availability

The datasets presented in this article are not readily available because the data are part of an ongoing study. Requests to access the datasets should be directed to the corresponding author.

## Supplementary Materials

The following supporting information can be downloaded at: https://www.mdpi.com/article/10.3390/audiolres15050113/s1: Table S1: Summary of studies analysing relationships between SSQ and hearing-in-noise tests, in non-cochlear-implanted participants. Table S2: Models concerning EEAS, EEAS*Noise*, and EEAS*Quiet*.

## Abbreviations

The following abbreviations are used in this manuscript:

Cis	Confidence intervals
EEAS	Extended Effort Assessment Scale scores (from 0 to 10 maximum effort)
EEAS*Noise*	Subscale “noise” of the Extended Effort Assessment Scale scores
EEAS*Quiet*	Subscale “quiet” of the Extended Effort Assessment Scale: items depicting listening situations in quiet
HA	Hearing aid
HA Experience	Total experience with hearing aids, in years
HA_PTA	Hearing-aided threshold (average of 500 Hz, 1, 2 and 4 kHz pure-tone threshold)
HA_SPIN	Hearing-aided speech-in-noise thresholds
HA_TLE	Speech-in-noise task-related listening effort threshold, obtained with hearing aids
HADW	Hearing aid daily wear duration, in hours
HINT	Hearing-in-Noise Test
MSE	Mean squared error
PROMs	Patient-related outcome measures
SD	Standard deviation
SE	Standard error
SPIN	Speech in cocktail party noise threshold
SSQ	Speech Spatial and Qualities of Hearing Scale; full scale
SSQ*Speech*	Subscale “Speech” of the SSQ
SSQ*Spatial*	Subscale “Spatial” of the SSQ
SSQ*Quality*	Subscale “Quality” of the SSQ
15iSSQ	Speech Spatial and Qualities of Hearing Scale; 15-item short form
15iSSQ*Speech*	Subscale “Speech” of the 15iSSQ, i.e., the first 5 items
15iSSQ*Spatial*	Subscale “Spatial” of the 15iSSQ, i.e., items 6 to 10
15iSSQ*Quality*	Subscale “Quality” of the SSQ, i.e., the last 5 items
TLE	Task-related listening effort threshold
UA	Unaided
UA_PTA	Unaided threshold (average of 500 Hz, 1, 2, and 4 kHz pure-tone thresholds)
UA_SPIN	Unaided speech-in-noise thresholds, obtained without hearing aids
UA_TLE	Speech-in-noise task-related listening effort threshold, obtained without hearing aids
